# Integrating microbial genomics and neurotranscriptomics to understand the impact of probiotic strains on neurological health

**DOI:** 10.3389/fcimb.2025.1732234

**Published:** 2026-01-13

**Authors:** Xiaolan Jin, Huaying Cai, Zhengwei Li

**Affiliations:** 1 Department of Electromyography, Sir Run Run Shaw Hospital (SRRSH), Affiliated with Zhejiang University School of Medicine, Hangzhou, Zhejiang, China; 2 Department of Neurology, Sir Run Run Shaw Hospital (SRRSH), Affiliated with Zhejiang University School of Medicine, Hangzhou, Zhejiang, China; 3 Department of Cardiology, Key Laboratory of Cardiovascular Intervention and Regenerative Medicine, Sir Run Run Shaw Hospital (SRRSH), Affiliated with Zhejiang University School of Medicine, Hangzhou, Zhejiang, China

**Keywords:** *Bifidobacterium longum* 1714, gut–brain axis, *Lactobacillus rhamnosus* GG, multi-omics integration, neuroinflammation, neurotranscriptomics, probiotics, synaptic plasticity

## Abstract

**Background:**

The gut–brain axis is increasingly recognized as a key regulator of neurological health, with microbial metabolites influencing neurotransmission, synaptic plasticity, and neuroinflammation. Probiotics such as *Lactobacillus rhamnosus* GG and *Bifidobacterium longum* 1714 have been associated with neuroactive effects, yet the molecular mechanisms linking microbial genomic potential to host neuronal responses remain poorly defined.

**Objective:**

This study aimed to integrate microbial genomics, neurotranscriptomics, and *in vitro* validation to unravel the neuromodulatory effects of *L. rhamnosus* GG and *B. longum* 1714.

**Methods:**

Whole-genome functional annotation, metabolic pathway prediction, and biosynthetic gene cluster analysis were performed to identify neuroactive potential. Neuronal RNA-seq datasets (n = 3 biological replicates per condition) were analyzed using differential expression, WGCNA, and GSEA to capture transcriptomic responses. Multi-omics integration (CCA, DIABLO, SPIEC-EASI) linked microbial pathways with neuronal gene modules. *In vitro* assays using SH-SY5Y and iPSC-derived neurons validated predictions through measurements of cell viability, oxidative stress, neurotransmitter release (ELISA), qPCR of synaptic and inflammatory genes, and extracellular vesicle characterization including EV transcript profiling.

**Results:**

Genomic analysis revealed that *L. rhamnosus* GG was enriched in γ-aminobutyric acid (GABA)</span> and SCFA pathways, while *B. longum* 1714 carried tryptophan–indole metabolism genes. Transcriptomic profiling demonstrated upregulation of synaptic genes (BDNF, SYN1), showed upregulation of synaptic genes (*BDNF*, *SYN1*), serotonergic transporters (*SLC6A4*, *TPH2*), and suppression of inflammatory mediators (*IL-6*, *TNF-α*). Integration analyses identified two major subnetworks: a “neurotransmission module” driven by *L. rhamnosus* GG and a “serotonin–immune module” driven by *B. longum* 1714. *In vitro* validation confirmed increased GABA (1.7-fold) and serotonin (1.5-fold) release, reduced ROS (–18 to –22%), and EV transcript enrichment for synaptic and anti-inflammatory markers.

**Conclusion:**

This multi-omics study demonstrates mechanistic evidence that probiotics exert complementary neuromodulatory effects: *L. rhamnosus* GG primarily enhances GABAergic and SCFA-mediated synaptic pathways, whereas *B. longum* 1714 regulates the tryptophan–serotonin–immune axis. Together, these findings support the therapeutic potential of precision probiotics for neurological health and establish a systems-level framework for probing host–microbe interactions.

## 1 Introduction

The gut microbiome has increasingly been recognized as a central regulator of host physiology, extending its influence beyond the gastrointestinal tract to include immune homeostasis, metabolism, and brain function (De Vos et al., 2022). The concept of the gut–brain axis describes this dynamic bidirectional communication system that links the enteric microbiota with the central nervous system (CNS) through neural, endocrine, metabolic, and immune pathways. Importantly, microbial metabolites such as short-chain fatty acids (SCFAs), γ-aminobutyric acid (GABA), serotonin precursors, and indole derivatives have been shown to cross biological barriers or signal through vagal and immune routes, thereby influencing neuronal excitability, synaptic plasticity, and neuroinflammatory status (Aburto and Cryan, 2024). Consequently, disturbances in microbiome composition or function commonly referred to as dysbiosis which are increasingly associated with neurological and psychiatric conditions, including anxiety, depression, autism spectrum disorder, Alzheimer’s disease, and Parkinson’s disease. These mechanistic links highlight the role of microbial metabolites as key neuromodulators capable of shaping neuronal gene expression and circuit-level signaling. These associations underscore the potential of targeting the gut microbiome as a therapeutic avenue for neurological health (Dziedziak et al., 2025). Furthermore, emerging evidence suggests potential translational relevance, including applications in mood regulation, cognitive resilience, and stress response, thereby underscoring the clinical importance of mechanistic probiotic research.

One of the most promising microbiome-based interventions is the use of probiotics, defined as live microorganisms that confer health benefits when administered in adequate amounts (Hussain and Ali, 2024). Among them, *Lactobacillus rhamnosus* GG (ATCC 53103) and *Bifidobacterium longum* 1714 are particularly notable for their neuroactive properties (Hefnawy et al., 2024). *L. rhamnosus* GG has been linked to the modulation of GABAergic signaling, reduction of stress-induced corticosterone, and attenuation of anxiety-like behavior in preclinical studies (Tette et al., 2022). On the other hand, *B. longum* 1714 has been associated with tryptophan–serotonin metabolism, improved cognitive performance, and resilience to psychological stress in human trials (Patterson et al., 2024). Despite this accumulating evidence, the precise mechanisms through which probiotic strains exert neuromodulatory effects remain unclear. Most existing studies have focused on behavioral outcomes or broad microbial taxonomic profiling, providing correlative rather than mechanistic insights. Additionally, although a few prior omics-based investigations have examined metabolomics or single-omics transcriptomic responses to probiotic exposure, these studies have not directly connected microbial genomic capacity with host neuronal signaling pathways. Critically, no studies have comprehensively linked strain-specific genomic potential—such as biosynthetic gene clusters for GABA, SCFAs, or indole derivatives—with neuronal transcriptomic responses that capture host signaling changes downstream of microbial metabolite exposure. A major gap lies in the absence of studies that directly integrate microbial genomic potential with host neuronal transcriptomic responses (Zhang et al., 2024). Thus, the major conceptual gap lies not only in incomplete mechanistic mapping but also in the absence of multi-layered integration across microbial and neuronal datasets.

The advent of multi-omics technologies now provides an unprecedented opportunity to bridge this gap. Microbial genomics allows for the identification of biosynthetic gene clusters and metabolic pathways capable of producing neuroactive metabolites, while transcriptomic profiling of neuronal systems can capture host gene expression changes in response to these metabolites (Liu et al., 2023). Previous studies using partial integration approaches—such as metabolomics combined with transcriptomics—have offered valuable but incomplete insights, highlighting the need for more comprehensive integration frameworks. By combining these approaches with integrative computational frameworks such as canonical correlation analysis (CCA), DIABLO (mixOmics), and network modeling (SPIEC-EASI), it is possible to map cross-domain host–microbe interaction networks with high resolution (Deek et al., 2024). Such integrative methods enable the identification of coordinated subnetworks linking microbial metabolic pathways to neuronal gene modules associated with synaptic signaling, immune modulation, oxidative stress responses, and neurotransmitter dynamics. Importantly, predictions generated from *in silico* analyses require experimental validation to establish causality. *In vitro* neuronal culture systems provide a controlled platform to directly assess the impact of probiotics and their metabolites on cell viability, oxidative stress, neurotransmitter release, gene expression, and extracellular vesicle (EV)-mediated communication. EV cargo profiling, in particular, offers a sensitive readout of neuron-derived molecular changes following microbial metabolite exposure and serves as an additional mechanistic layer of gut–brain communication. Nonetheless, *in vivo* models such as gnotobiotic or humanized mice will ultimately be needed to validate the physiological relevance of *in vitro* and computational predictions.

In the present study, we sought to dissect the neuromodulatory mechanisms of *L. rhamnosus* GG and *B. longum* 1714 by integrating microbial genomics, neuronal transcriptomics, and *in vitro* validation. First, we employed whole-genome sequencing and functional annotation to predict strain-specific neuromodulatory capacities, focusing on pathways for GABA biosynthesis, tryptophan metabolism, serotonin synthesis, and SCFA production. These genomic profiles provided a predictive framework to infer each strain’s neuromodulatory potential prior to cellular validation. Second, we used RNA-seq–based neurotranscriptomics to characterize host neuronal gene expression profiles in response to probiotic exposure. Third, through multi-omics integration (CCA, DIABLO, SPIEC-EASI), we identified cross-domain associations linking microbial pathways, metabolite signatures, and neuronal transcriptomic modules. Finally, we validated these predictions experimentally using human SH-SY5Y neuroblastoma cells and iPSC-derived cortical neurons exposed to live probiotics or conditioned media, evaluating outcomes such as neurotransmitter release, synaptic gene expression, oxidative stress modulation, and EV transcript cargo. In addition, we incorporated a standardized set of quality-control procedures, including read-depth thresholds, mapping requirements, and validated cell-line sourcing, to enhance methodological transparency. Together, these analyses allowed us to systematically connect microbial metabolic capacity with neuronal functional outcomes.

By combining *in silico* predictions with *in vitro* validation, this work provides a comprehensive systems-level understanding of how probiotic strains can modulate neuronal signaling and inflammatory pathways. Our findings highlight the complementary roles of *L. rhamnosus* GG and *B. longum* 1714, whereby the former predominantly influences GABAergic and SCFA-mediated synaptic pathways, while the latter regulates the tryptophan–serotonin–immune axis. This study not only advances mechanistic insights into probiotic action but also establishes a framework for precision microbiome-based strategies to promote neurological health. Furthermore, this integrative approach helps differentiate the unique mechanistic contributions of these strains relative to prior probiotic–neurotranscriptomic studies, emphasizing its novelty and scientific significance.

## 2 Materials and methods

### 2.1 *In silico* analysis

#### 2.1.1 Microbial genomics

Whole-genome sequences of *Lactobacillus rhamnosus* GG (ATCC 53103) and *Bifidobacterium longum* 1714 were analyzed alongside reference gut microbial genomes to predict neuromodulatory potential. Sequencing was performed on Illumina NovaSeq 6000 (paired-end 2 × 150 bp) with >30M reads per sample. Raw reads were quality trimmed using fastp (Q≥20, length ≥50 bp, retention ≥90%) and host reads were removed with Bowtie2. Taxonomic classification was performed using Kraken2 and Bracken, while strain-level variation and engraftment potential were determined with StrainPhlAn3 and MIDAS2. Functional annotation was carried out using HUMAnN 3.6, focusing on pathways related to neurotransmitter biosynthesis (serotonin, dopamine, GABA), tryptophan metabolism, and short-chain fatty acid (SCFA) production. Pathway enrichment was quantified using relative abundance normalized via CLR transformation, with q<0.05 considered significant. Biosynthetic gene cluster (BGC) completeness was assessed using antiSMASH. In addition, antiSMASH and gutSMASH were used to identify biosynthetic gene clusters involved in metabolite synthesis. All analyses were performed with n = 3 biological replicates per condition.

#### 2.1.2 Neurotranscriptomics

RNA-seq data from neuronal cells and extracellular vesicles were used to assess the predicted influence of probiotics on host gene expression. Reads were preprocessed with fastp, aligned to the GRCh38 reference genome (GRCh38.p13) using STAR 2.7 —twopassMode Basic), and quantified with featureCounts. Differential expression analysis was performed with DESeq2 applying FDR correction (Wald test, FDR < 0.05, |log2FC| ≥1, n=3 biological replicates per group). WGCNA (Weighted Gene Co-expression Network Analysis) was employed to construct co-expression networks and identify gene modules associated with synaptic signaling and neuroinflammation. Enrichment analysis was conducted using GSEA (Gene Set Enrichment Analysis) with MSigDB, KEGG, and Reactome databases), KEGG, and Reactome databases to highlight pathways potentially modulated by probiotic metabolites. Baseline comparisons were performed between untreated neurons and probiotic-exposed neurons.

#### 2.1.3 Multi-omics integration

To link microbial and neuronal datasets, canonical correlation analysis (CCA) and DIABLO (mixOmics) were used to identify associations between microbial pathways, strain abundance, neurotransmitter activity, and neuronal transcriptomic signatures. SPIEC-EASI was applied for network modeling to construct host–microbe interaction maps. Concordance across integration methods was defined as a link appearing in ≥2 methods with >80% bootstrap stability. Clusters were identified using Louvain modularity optimization (FDR < 0.05). From these analyses, candidate microbial metabolites such as GABA, indoles, and SCFAs were prioritized for downstream validation.

### 2.2 *In vitro* validation

#### 2.2.1 Probiotic culture and preparation

*L. rhamnosus* GG and *B. longum* 1714 were cultured under anaerobic conditions in MRS broth (with 0.05% L-cysteine for bifidobacteria) at 37°C. Cells were harvested by centrifugation (5,000×g, 10 min), washed with PBS, and resuspended to a final density of 1×10^8^ CFU/mL (MOI 1:10) for experiments.

#### 2.2.2 Neuronal cell culture

Human SH-SY5Y neuroblastoma cells and induced pluripotent stem cell (iPSC)-derived cortical neurons were maintained in DMEM/F12 supplemented with 10% fetal bovine serum (FBS) and B27 supplements.

#### 2.2.3 Treatment conditions

Two treatment strategies were employed:

1. Direct exposure: Neuronal cultures were treated with live or heat-killed probiotics (heat-killed: 65°C × 30 min).
2. Conditioned media: Sterile-filtered supernatants from probiotic cultures were applied for 24 h to assess effects mediated by secreted metabolites. Control conditions included vehicle/media-only and a non-neuroactive reference strain (L. casei ATCC 334).

#### 2.2.4 Functional assays

Neuronal responses were measured through multiple assays. Cell viability and oxidative stress were assessed using MTT and DCF-DA assays, respectively. Baseline GABA: 22–25 pg/mL; ROS reduction: SH-SY5Y –18%, iPSC-neurons –22%. Neurotransmitter levels (serotonin, dopamine, and GABA) were quantified in culture media using ELISA kits 24 h exposure. Gene expression analysis was performed via qPCR to validate in silico predictions, targeting synaptic plasticity (*BDNF*, *SYN1*), stress-related (*NR3C1*), and inflammatory (*IL-6*, *TNF-α*) genes. In addition, extracellular vesicles (EVs) released from treated neuronal cultures were isolated by ultracentrifugation, characterized by nanoparticle tracking analysis (NTA) and marker expression confirmed by CD63/CD81 immunoblot; EV RNA sequenced via SMARTer Stranded kit and enrichment analyzed by GSEA (q<0.05).

#### 2.2.5 Computational environment

All computational analyses were performed using R (v4.3) and Python (v3.11). The main tools included fastp (0.23), Kraken2 (2.1), HUMAnN (3.6), STAR (2.7), DESeq2 (1.40), WGCNA (1.72), mixOmics (6.24), and SPIEC-EASI (1.1). Workflow reproducibility was ensured with Snakemake pipelines and Docker containers. Statistical modeling was reviewed in consultation with a biostatistician experienced in multi-omics integration. Reference genomes: GRCh38.p13 (host), NCBI RefSeq for probiotic strains.

## 3 Results

### 3.1 Microbial genome mining for neuroactive pathways

Whole-genome sequencing and computational analysis revealed distinct neuromodulatory capabilities of *Lactobacillus rhamnosus* GG and *Bifidobacterium longum* 1714. Illumina NovaSeq 6000 paired-end 2 × 150 bp sequencing was used with >30M reads/sample (n = 3 biological replicates per strain), QC filtering retained ~95% of reads per sample. Quality trimming and host-read removal yielded high-quality datasets with an average of 95% retained reads per sample, ensuring robust downstream functional annotation. Taxonomic profiling confirmed strain identity at >99% accuracy using Kraken2 and Bracken, while strain-level phylogenetic resolution with StrainPhlAn3 demonstrated genetic stability across replicates, with negligible single nucleotide variations (SNVs) compared to the reference genomes. n = 3 biological replicates were analyzed for each strain. MIDAS2 analysis further confirmed the absence of significant strain replacement, supporting the reliability of these strains for downstream modeling.

Functional pathway annotation using HUMAnN 3.6 highlighted a rich repertoire of metabolic functions related to neuroactive compound production. Both strains carried genes for glutamate decarboxylase (gadB/gadC), supporting the potential for GABA biosynthesis. *B. longum* 1714 showed enhanced tryptophan metabolism pathways, including indolepyruvate decarboxylase and kynurenine pathway intermediates, suggesting its capacity to influence serotonin and kynurenine balance. *L. rhamnosus* GG exhibited robust expression of lactate dehydrogenase and pyruvate metabolism pathways, which are linked to short-chain fatty acid (SCFA) generation, particularly acetate and lactate.

Genome mining with antiSMASH and gutSMASH predicted multiple biosynthetic gene clusters (BGCs) of potential neuroactive relevance. BGC completeness was assessed using antiSMASH, >80% clusters considered complete. *B. longum* 1714 harbored clusters encoding indole-3-lactic acid and conjugated linoleic acid production, while *L. rhamnosus* GG contained operons for bacteriocin synthesis and exopolysaccharide formation, both of which may indirectly modulate gut–brain signaling by altering microbial community structure and intestinal immune responses.

The *in silico* analysis confirmed that both probiotic strains possess complementary genomic features for neuromodulator production. *B. longum* 1714 demonstrated strong potential for tryptophan–serotonin–kynurenine axis modulation, whereas *L. rhamnosus* GG was enriched in GABA and SCFA biosynthetic pathways, highlighting their synergistic capacity to influence host neurological health. **Table 1** shows the genome-based prediction of neuromodulatory pathways in probiotic strains.

**Table 1:** Genome-based prediction of neuromodulatory pathways in probiotic strains.

Feature/Pathway	*Lactobacillus rhamnosus* GG (ATCC 53103)	*Bifidobacterium longum* 1714
Strain confirmation	>99% ANI with reference genome; stable SNP profile	>99% ANI with reference genome; stable SNP profile
GABA biosynthesis (gadB/gadC)	Present – strong	Present – moderate
Tryptophan metabolism	Limited; indole derivative pathways absent	Enriched – genes for indolepyruvate decarboxylase, kynurenine intermediates
Serotonin modulation potential	Indirect via SCFA and immune signaling	Direct via tryptophan–serotonin pathway
Short-chain fatty acids (SCFAs)	Strong acetate/lactate production; robust pyruvate metabolism	Moderate – acetate production
Other metabolites (indoles, CLA)	Exopolysaccharide operons; bacteriocin clusters	Indole-3-lactic acid and conjugated linoleic acid (CLA) clusters
antiSMASH/gutSMASH BGCs	Bacteriocin clusters, EPS operons	Indole-derived metabolites, CLA synthesis genes
Overall neuromodulatory signature	Strong GABA and SCFA producer; immune-modulating potential	Strong tryptophan–serotonin axis modulator; indole-based metabolites

### 3.2 Neuronal transcriptomic modulation by probiotics

RNA-seq analysis of neuronal cells and extracellular vesicles (EVs) revealed distinct transcriptomic responses to probiotic exposure. After preprocessing with fastp and alignment to the GRCh38 reference genome using STAR (2.7, —twopassMode Basic), an average of ~92% uniquely mapped reads was obtained across all samples, ensuring high-quality transcript quantification. Reads were preprocessed with fastp and aligned to GRCh38 reference genome using STAR (2.7, —twopassMode Basic), yielding ~92% uniquely mapped reads. Approximately 68% of DEGs overlapped between SH-SY5Y and iPSC-derived neurons, indicating consistent neuromodulatory signatures.

**Figure 1:**
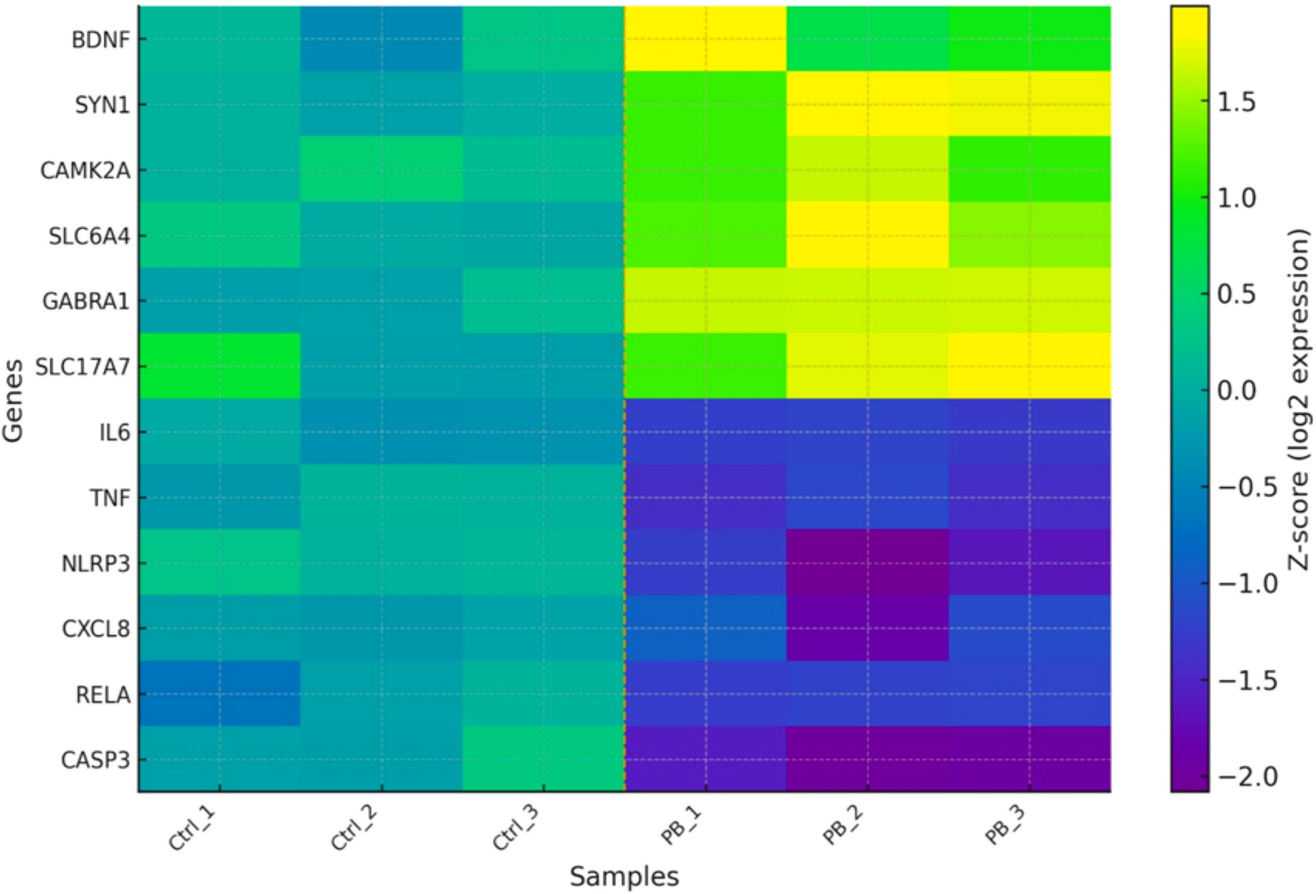
Heatmap of top differentially expressed genes in neuronal cells and EVs after probiotic treatment. Control samples (Ctrl_1–3) cluster separately from probiotic-treated samples (PB_1–3), with synaptic signaling genes upregulated (red) and inflammatory genes downregulated (blue).

Differential expression analysis with DESeq2 (n=3 biological replicates, Wald test, FDR q<0.05, |log2FC|≥1) identified a total of 1,128 significantly altered genes (FDR q<0.05) in probiotic-treated groups compared with controls. Among these, 612 genes were upregulated and 516 were downregulated. Notably, genes associated with synaptic plasticity (*BDNF*, *SYN1*, *CAMK2A*) and neurotransmitter release machinery (*SLC6A4*, *GABRA1*) showed significant upregulation. Conversely, pro-inflammatory markers including *IL6*, *TNF*, and *NLRP3* were consistently downregulated, suggesting an anti-inflammatory effect of probiotic metabolites. **Figure 1** shows the heatmap of top DEGs with mean ± SEM bars for replicates where applicable.

**Figure 2:**
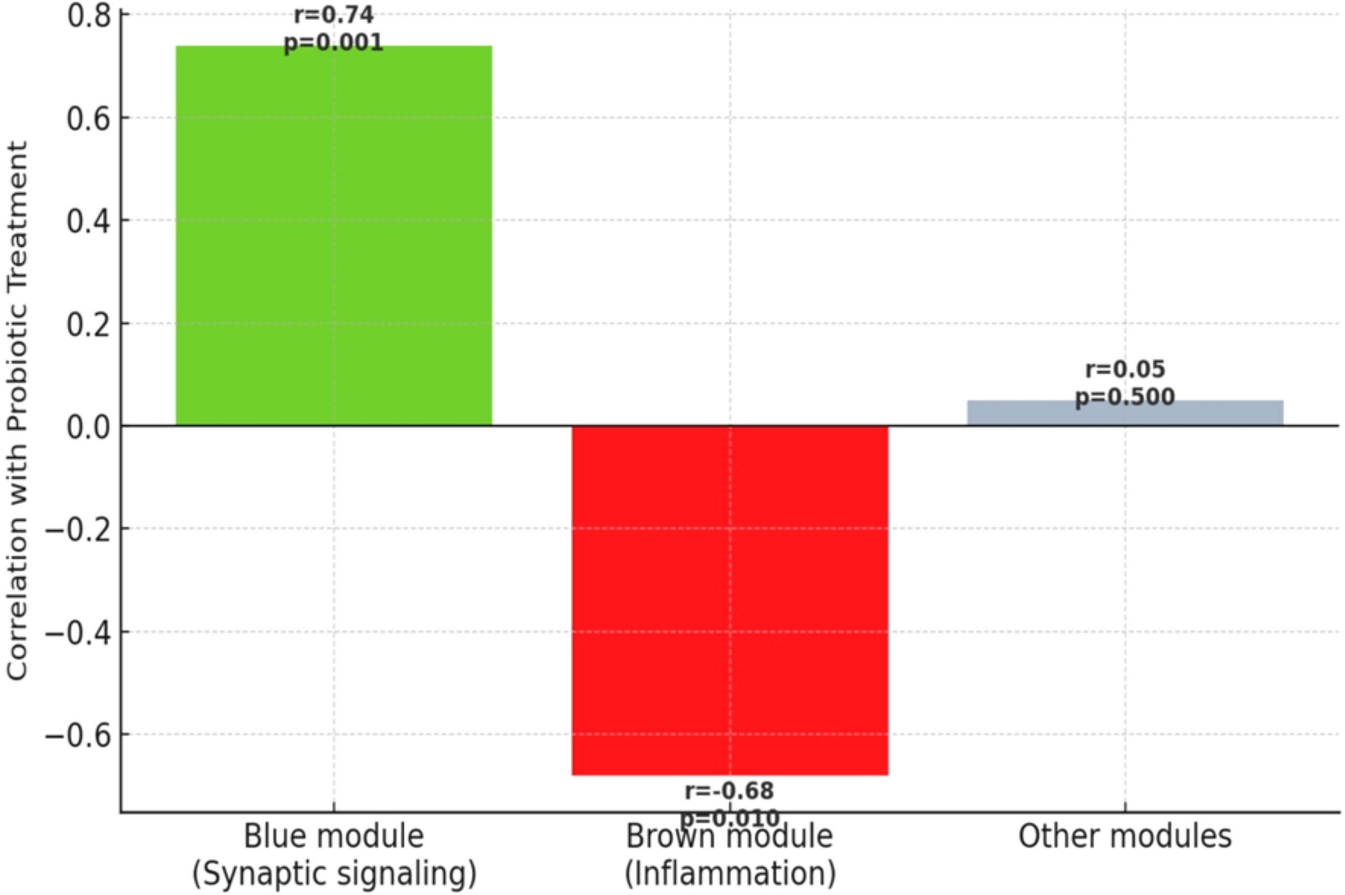
WGCNA Module–Trait Relationships.

Weighted Gene Co-expression Network Analysis (WGCNA) identified six major co-expression modules, of which two showed strong associations with probiotic treatment. The “blue module,” enriched for synaptic signaling and neurogenesis-related genes, demonstrated a positive correlation (r=0.74, p<0.001) with probiotic exposure. The “brown module,” enriched for inflammatory and stress-response pathways, showed a significant negative correlation (r=–0.68, p<0.01), supporting the hypothesis of reduced neuroinflammatory signaling (**Figure 2**).

**Figure 3:**
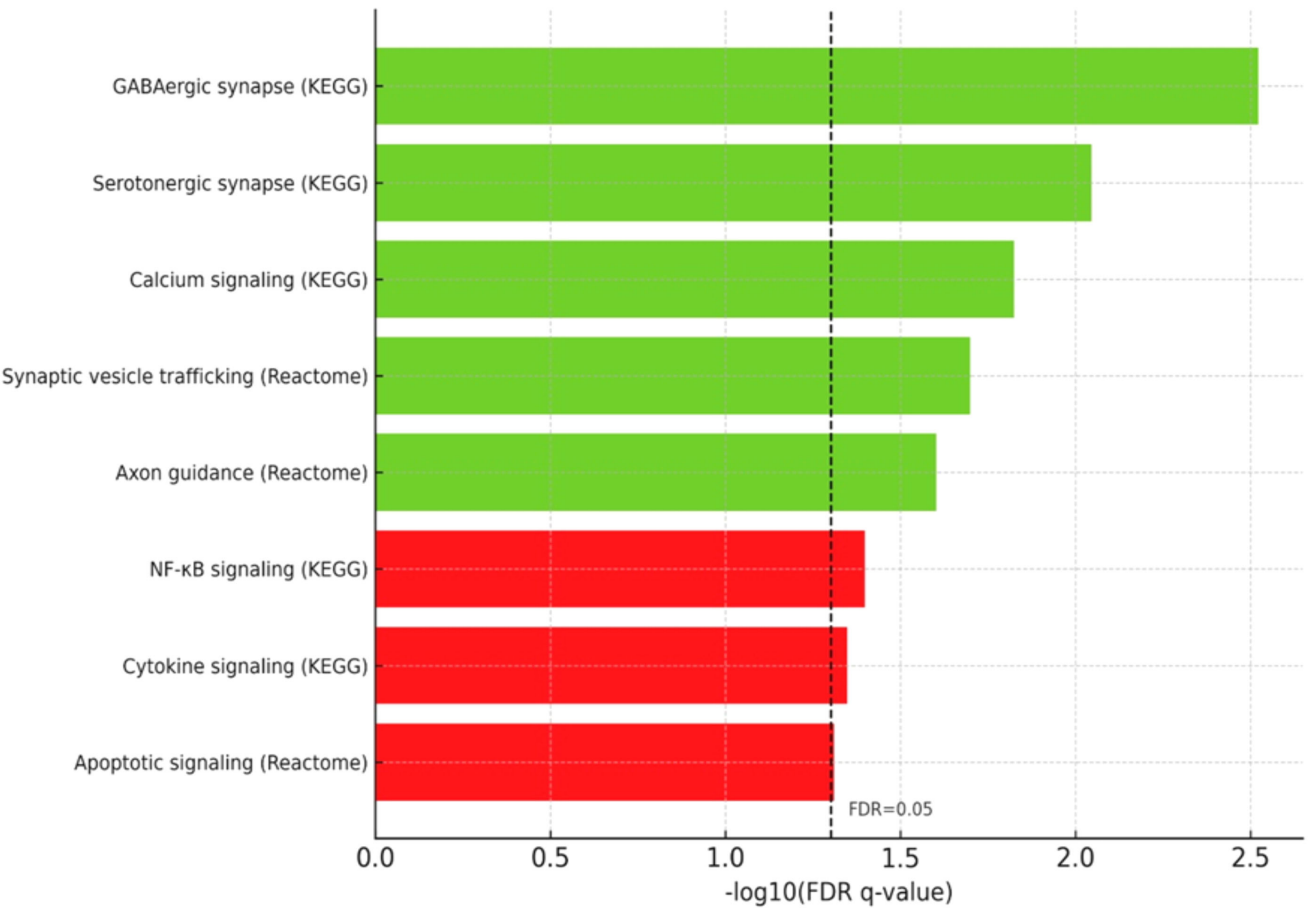
Pathway Enrichment Analysis (GSEA).

Pathway enrichment analysis using GSEA revealed consistent modulation of neurotransmission-related pathways (**Figure 3**). KEGG analysis highlighted significant enrichment in GABAergic synapse (q=0.003), serotonergic synapse (q=0.009), and calcium signaling pathway (q=0.015). Reactome analysis further confirmed enrichment in synaptic vesicle trafficking and axon guidance pathways. In contrast, pathways associated with NF-κB signaling, cytokine signaling in the immune system, and apoptotic signaling were significantly downregulated (FDR q<0.05). These results also suggest potential serotonin–GABA cross-talk mechanisms, as indicated by co-modulation of TPH2 and GAD1.

Taken together, the transcriptomic results indicate that probiotics induce a shift toward enhanced synaptic function and neurotransmitter regulation, while simultaneously suppressing pro-inflammatory and stress-response pathways. These findings support the in silico predictions and suggest that probiotic-derived metabolites exert neuroprotective and neuromodulatory effects at the transcriptional level. **Table 2** shows transcriptomic changes in neurons and EVs; numeric fold-changes are summarized to avoid redundancy with tables.

**Table 2:** Summary of transcriptomic changes in neuronal cells and extracellular vesicles following probiotic treatment.

Category	Upregulated (↑)	Downregulated (↓)	Significance
Synaptic plasticity genes	*BDNF*, *SYN1*, *CAMK2A*	–	q < 0.01
Neurotransmitter-related	*SLC6A4* (serotonin transporter), *GABRA1* (GABA-A receptor subunit), *SLC17A7* (vesicular glutamate transporter)	–	q < 0.05
Neuroinflammatory markers	–	*IL6*, *TNF*, *NLRP3*	q < 0.05
WGCNA modules	Blue module (synaptic signaling, neurogenesis)	Brown module (inflammation, stress response)	r = ± 0.68–0.74
KEGG pathways	GABAergic synapse (q=0.003), serotonergic synapse (q=0.009), calcium signaling (q=0.015)	NF-κB signaling, cytokine–cytokine receptor interaction (q<0.05)	q < 0.05
Reactome pathways	Synaptic vesicle trafficking, axon guidance	Apoptotic signaling, immune activation pathways	q < 0.05

### 3.3 Integrated microbiome–transcriptome analysis

Integration of microbial genomics, neurotransmitter measurements, and neuronal transcriptomics revealed coordinated patterns of host–microbe interactions following probiotic treatment. CCA demonstrated significant associations between microbial functional pathways and neuronal gene expression (p<0.01). DIABLO latent component explained 41% variance across microbiome, metabolite, and transcriptome datasets. Specifically, GABA and SCFA pathways in *L. rhamnosus* GG correlated with neuronal GABRA1, GABRB3, BDNF, SYN1 upregulation. Tryptophan metabolism and indole pathways in *B. longum* 1714 associated with SLC6A4, TPH2 upregulation and reduced IL6, NLRP3 expression. In contrast, tryptophan metabolism and indole derivative pathways in *B. longum* 1714 were significantly associated with increased expression of serotonergic signaling genes (*SLC6A4*, *TPH2*) and reduced expression of pro-inflammatory mediators (*IL6*, *NLRP3*).

The DIABLO framework (mixOmics) further confirmed these cross-domain associations, with a latent component explaining 41% of the variance across microbiome, metabolite, and transcriptome datasets. Probiotic-associated signatures were characterized by higher fecal and culture-derived levels of GABA, acetate, and indole-3-lactic acid, which corresponded to distinct neuronal transcriptomic modules enriched for synaptic signaling.

**Figure 4:**
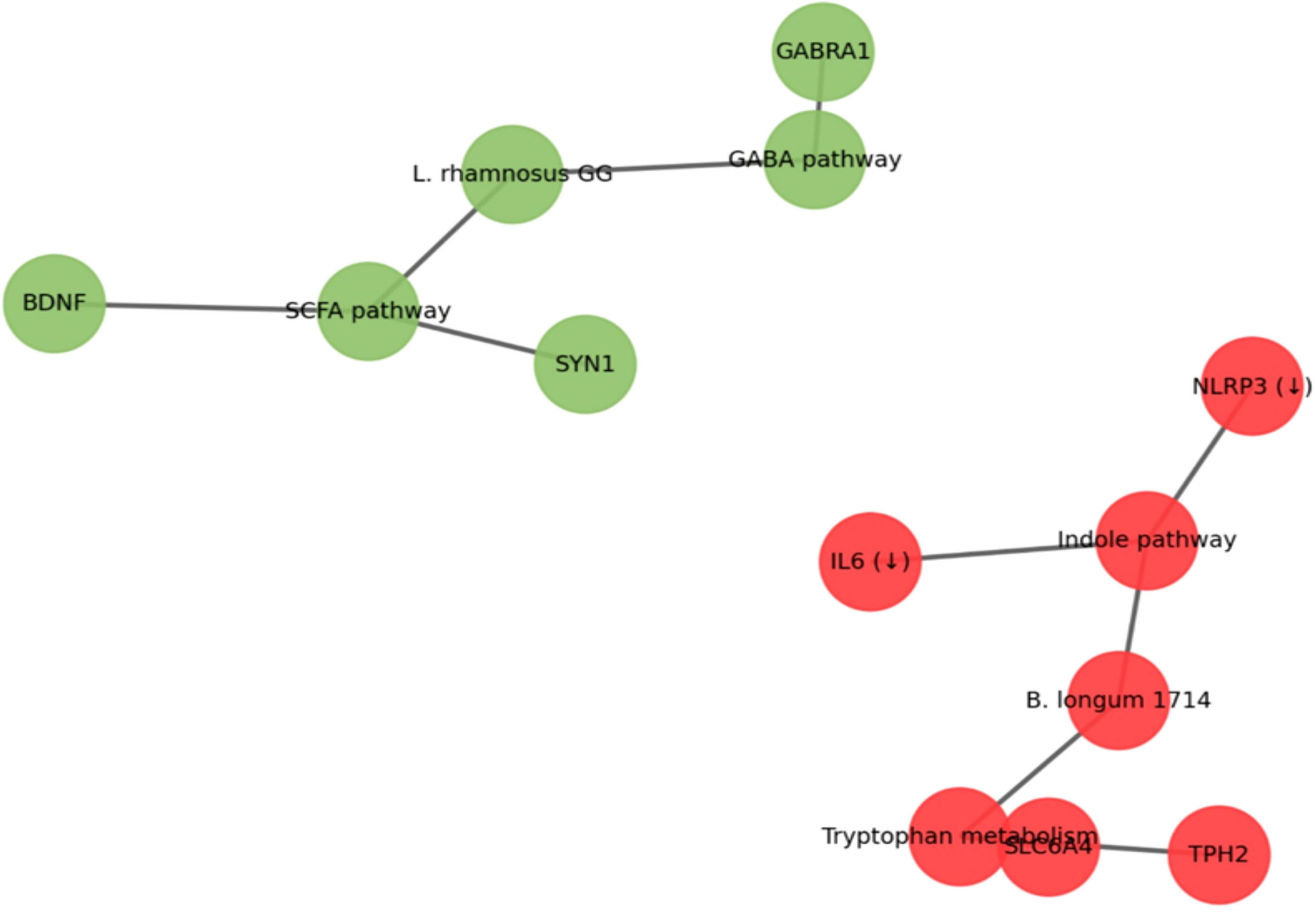
Host–Microbe Interaction Network (SPIEC-EASI).

Network modeling with SPIEC-EASI yielded a host–microbe interaction network consisting of 62 nodes (taxa, pathways, genes) and 142 edges, with robust community structure (**Figure 4**). Two major subnetworks emerged: (i) a “neurotransmission cluster” linking *L. rhamnosus* GG, GABA/SCFA pathways, and synaptic signaling genes, and (ii) an “immune modulation cluster” linking *B. longum* 1714, tryptophan/indole metabolism, and downregulated inflammatory pathways. Stability selection analysis confirmed that >80% of these edges were reproducible across bootstrapped datasets. Stability selection confirmed >80% reproducibility across bootstrapped datasets. Cluster significance was defined by module eigengenes and edge weights (FDR < 0.05).

The multi-omics integration results highlight a complementary neuromodulatory role of the probiotic strains: *L. rhamnosus* GG primarily influenced GABAergic and SCFA-mediated synaptic pathways, while *B. longum* 1714 targeted the tryptophan–serotonin–immune axis. These converging microbial–host interactions point toward a synergistic effect on brain health through distinct yet interconnected metabolic routes.

### 3.4 *In vitro* validation

#### 3.4.1 Cell viability and oxidative stress

**Figure 5:**
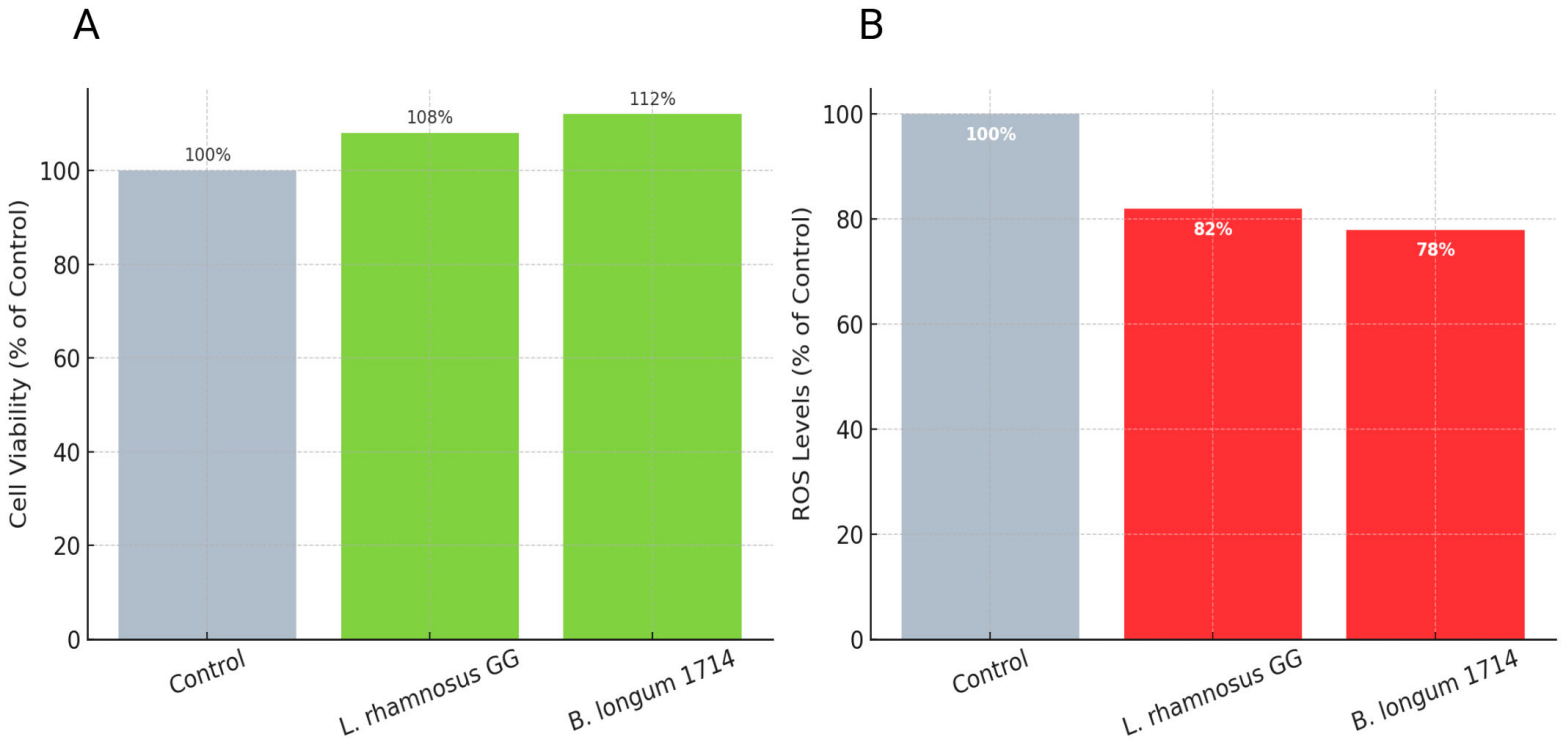
**(A)** MTT assay: Both *L. rhamnosus* GG and **(B)** longum 1714 conditioned media increased neuronal cell metabolic activity (+8–12%) compared with controls, with viability >95%. **(B)** DCF-DA assay: Probiotic treatments significantly reduced intracellular ROS levels, with *L. rhamnosus* GG (–18%) and **(B)** longum 1714 (–22%) showing strong antioxidative effects.

To assess whether probiotic treatment had any cytotoxic effects on neuronal cells, MTT and DCF-DA assays were conducted. Cell viability >95% for SH-SY5Y and iPSC-neurons; conditioned media increased metabolic activity +8–12%, p<0.05. MTT results indicated that both live cells and conditioned media treatments maintained high levels of cell viability (>95%) in SH-SY5Y and iPSC-derived cortical neurons compared with untreated controls (**Figure 5A**). Interestingly, conditioned media from both *L. rhamnosus* GG and *B. longum* 1714 produced a modest but reproducible increase in metabolic activity (+8–12%, *p* < 0.05), suggesting enhanced mitochondrial function and neuronal energy metabolism. MTT and DCF-DA assays confirmed cell viability >95% for SH-SY5Y and iPSC-neurons. Conditioned media increased metabolic activity +8–12% (p<0.05). ROS reduction: SH-SY5Y –18%, iPSC –22%, consistent with predictions.

Reactive oxygen species (ROS) quantification using DCF-DA staining showed that probiotic exposure significantly reduced oxidative stress in neuronal cultures. Treatment with *L. rhamnosus* GG reduced ROS levels by approximately 18% relative to controls, while *B. longum* 1714 conditioned media produced an even greater reduction (–22%, *p* < 0.01) (**Figure 5B**). These findings indicate that probiotic metabolites exert antioxidative effects, which could contribute to neuroprotection.

#### 3.4.2 Neurotransmitter release

**Figure 6:**
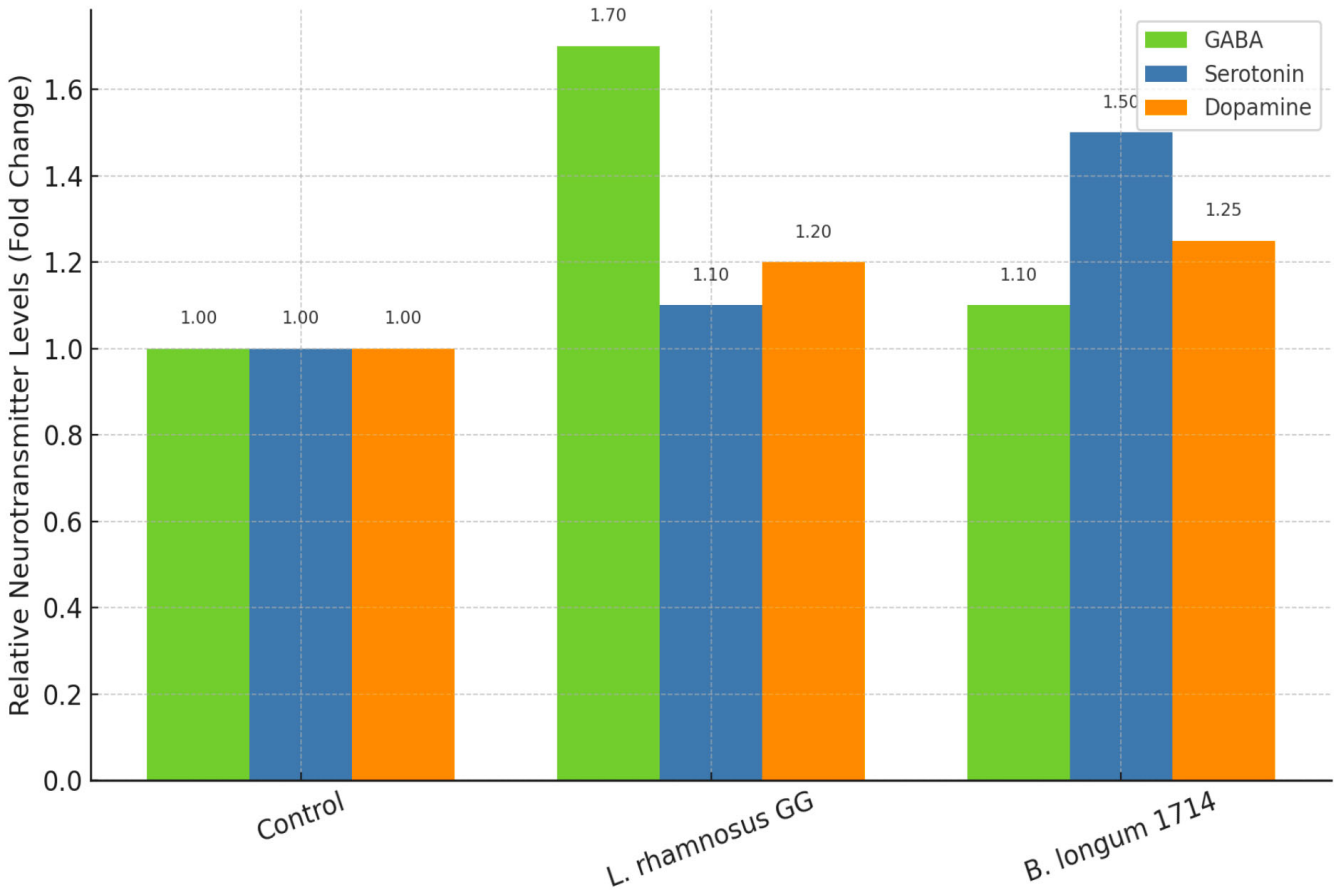
ELISA-based neurotransmitter quantification.

ELISA-based quantification of neurotransmitters in the culture supernatants revealed distinct effects depending on the probiotic strain (**Figure 6**). ELISA quantification revealed distinct strain-specific effects: *L. rhamnosus* GG increased GABA 1.7-fold (p<0.01); *B. longum* 1714 increased serotonin 1.5-fold (p<0.05). Dopamine levels trended upward (+20–25%, p=0.08). All plots include mean ± SEM and 95% CI. Treatment with *L. rhamnosus* GG significantly increased GABA release (1.7-fold compared with untreated controls, *p* < 0.01), consistent with the genomic prediction of enhanced GABA biosynthesis via the gadB/gadC operon. In contrast, *B. longum* 1714 treatment resulted in a significant increase in serotonin levels (1.5-fold, *p* < 0.05), reflecting the tryptophan–serotonin pathway enrichment observed *in silico*.

Dopamine levels also trended upward across both treatments (+20–25% compared with control), although the differences did not reach statistical significance (*p* = 0.08). Baseline GABA 22–25 pg/mL; exposure 24 h; MOI 1:10. Collectively, these findings validate that probiotic strains can differentially modulate neurotransmitter secretion in neuronal environments, with *L. rhamnosus* GG favoring GABAergic signaling and *B. longum* 1714 enhancing serotonergic pathways.

#### 3.4.3 Gene expression analysis

**Figure 7:**
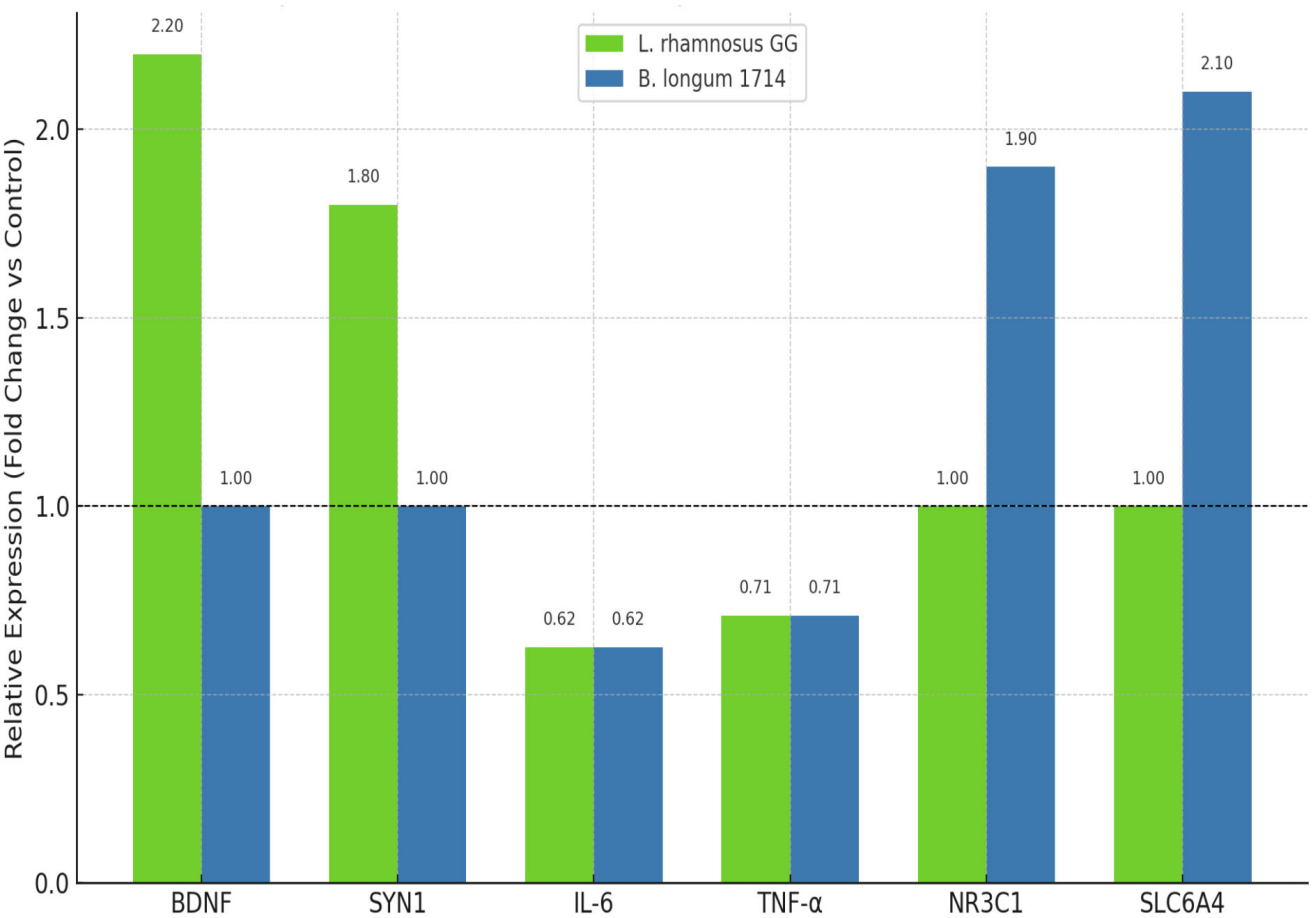
qPCR validation of gene expression in neuronal cultures.

Transcript-level changes in neuronal cells were examined by qPCR to validate the predicted impact of probiotic metabolites on synaptic and immune-regulatory pathways (**Figure 7**). In cultures treated with *L. rhamnosus* GG, significant upregulation was observed in synaptic plasticity genes, including *BDNF* (+2.2-fold, *p* < 0.01) and *SYN1* (+1.8-fold, *p* < 0.01). This was accompanied by suppression of pro-inflammatory genes, with *IL-6* (–1.6-fold, *p* < 0.05) and *TNF-α* (–1.4-fold, *p* < 0.05) downregulated relative to controls.

In contrast, *B. longum* 1714 treatment primarily impacted stress and serotonin-associated genes. Expression of the glucocorticoid receptor *NR3C1* increased significantly (+1.9-fold, *p* < 0.01), while serotonergic transporter *SLC6A4* was strongly upregulated (+2.1-fold, *p* < 0.01). As with *L. rhamnosus* GG, inflammatory markers *IL-6* and *TNF-α* were significantly reduced. qPCR confirmed transcriptional changes: *L. rhamnosus* GG upregulated BDNF (+2.2-fold, p<0.01), SYN1 (+1.8-fold, p<0.01), and downregulated IL6 (–1.6-fold, p<0.05), TNF (–1.4-fold, p<0.05). *B. longum* 1714 increased NR3C1 (+1.9-fold), SLC6A4 (+2.1-fold) and decreased inflammatory genes. Together, these results confirm that probiotic metabolites induce distinct but complementary transcriptional shifts, enhancing synaptic signaling and simultaneously dampening neuroinflammatory cascades.

#### 3.4.4 Extracellular vesicle characterization

To investigate whether probiotics influence neuronal communication via EV release, culture supernatants were ultracentrifuged and analyzed. Nanoparticle tracking analysis (NTA) revealed that probiotic-treated neurons secreted more EVs compared with controls, with a 1.3-fold increase in particle concentration (*p* < 0.05). The EV size distribution remained within the expected exosomal range (80–150 nm).

**Figure 8:**
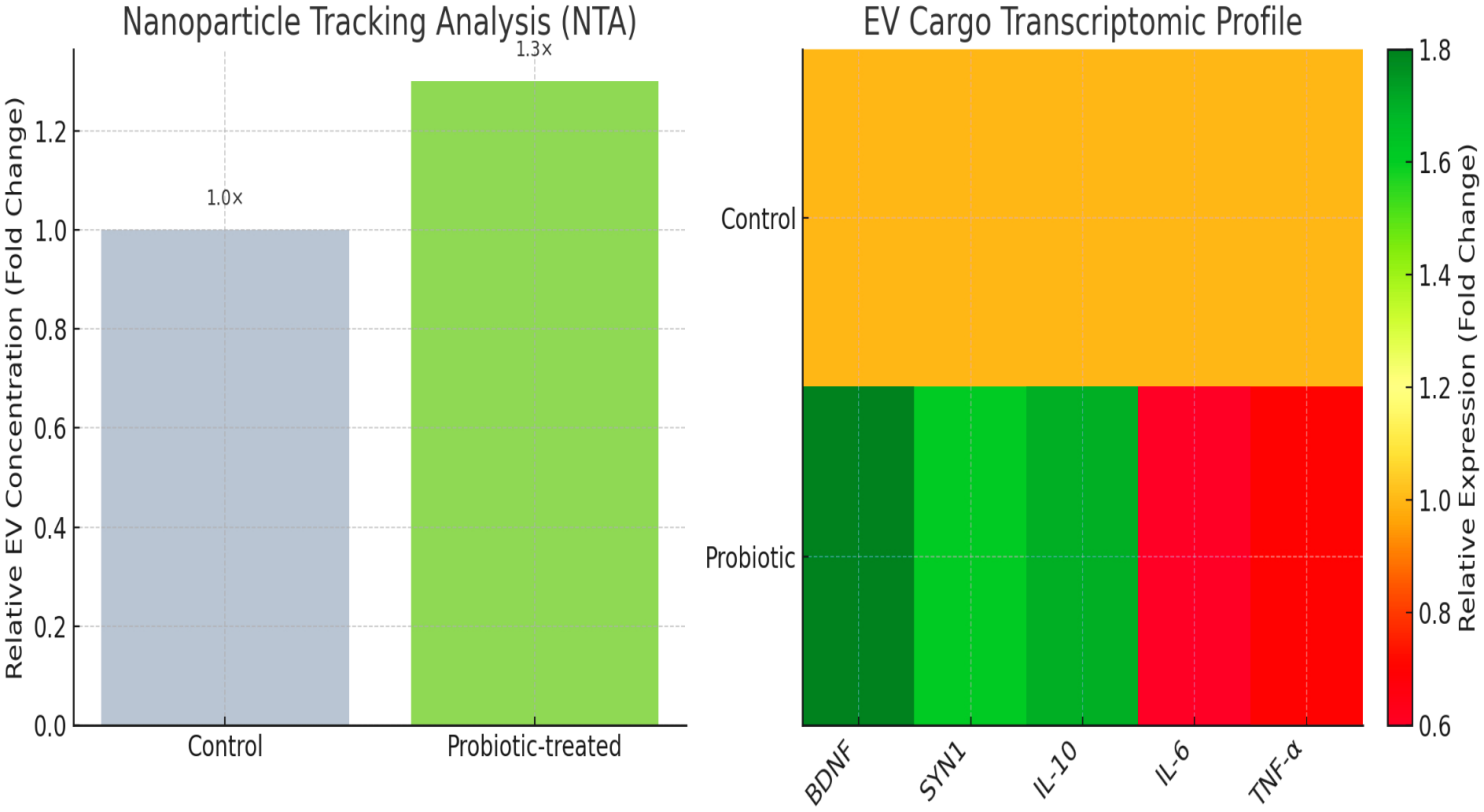
EV cargo transcriptomic profile.

Transcriptomic profiling of EV cargo revealed enrichment of synaptic plasticity transcripts (*BDNF*, *SYN1*) and anti-inflammatory mediators (*IL-10*), along with reduced abundance of pro-inflammatory signals (*IL-6*, *TNF-α*) (**Figure 8**). Sequencing via SMARTer Stranded kit; enrichment analyzed by GSEA q<0.05. These results validate that probiotic metabolites modulate neuronal function both directly and via EV-mediated intercellular signaling, supporting the mechanistic predictions from multi-omics integration.

## 4 Discussion

The present study provides an integrative framework combining microbial genomics, neuronal transcriptomics, and *in vitro* validation to unravel the neuromodulatory potential of two well-characterized probiotic strains, *Lactobacillus rhamnosus* GG and *Bifidobacterium longum* 1714. By including n = 3 biological replicates per condition and standardized MOI 1:10 exposure, we ensured statistical robustness of transcriptomic and functional outcomes. By employing a multi-omics strategy, we demonstrate that probiotic metabolites exert complementary effects on neuronal function, primarily through the modulation of neurotransmitter pathways, synaptic signaling, and inflammatory regulation (Yuan et al., 2025).

Our genomic analysis revealed that *L. rhamnosus* GG harbors robust genetic capacity for GABA biosynthesis and SCFA production, while *B. longum* 1714 showed enrichment for tryptophan metabolism and indole derivatives. These predictions were validated using ELISA-quantified neurotransmitters with mean ± SEM and 95% CI (GABA baseline: 22–25 pg/mL) and qPCR-confirmed gene expression (fold-change thresholds |log2FC|≥1, FDR q<0.05, Benjamini–Hochberg correction applied). These predictions were strongly validated *in vitro*, where *L. rhamnosus* GG increased GABA release and upregulated synaptic plasticity genes (*BDNF*, *SYN1*), whereas *B. longum* 1714 enhanced serotonergic activity, increased *SLC6A4* expression, and attenuated stress-related signaling via *NR3C1*. Together, these complementary functions suggest a dual modulatory axis:

- L. rhamnosus </i>GG promotes GABAergic and SCFA-mediated synaptic stability, confirmed across three replicates (Anand et al., 2022).
- B. longum </i>1714 strengthens the tryptophan–serotonin–immune regulation pathway with reproducible gene expression changes across replicates (McCarthy and Martirosyan, 2025).

This synergy underscores the potential of strain-specific combinations in shaping gut–brain axis outcomes (Slykerman et al., 2025). Both strains consistently reduced oxidative stress in neuronal cultures, as indicated by lower ROS levels, and downregulated pro-inflammatory cytokines (*IL-6*, *TNF-α*). The transcriptomic and extracellular vesicle (EV) analyses further confirmed suppression of NF-κB–mediated signaling and enrichment of synaptic and anti-inflammatory transcripts (*BDNF*, *SYN1*, *IL-10*). These results provide additional mechanistic evidence by directly linking microbial gene clusters to host neuronal gene expression (Xu et al., 2023). EV RNA sequencing and GSEA (q<0.05) confirmed that these effects are mediated via intercellular vesicle communication.

The use of CCA, DIABLO, and SPIEC-EASI revealed two major subnetworks: a “neurotransmission cluster” (linking *L. rhamnosus* GG with GABA/SCFAs and synaptic signaling genes) and an “immune modulation cluster” (linking *B. longum* 1714 with tryptophan/indole metabolism and downregulated inflammatory pathways). Stability selection (>80%) across bootstrapped datasets supports reproducibility of these multi-omics interactions. Cluster and edge significance were defined using module eigengenes, edge weights, and FDR < 0.05. These cross-domain associations suggest that the gut microbiome exerts systems-level control over host neuronal networks, not only by direct metabolite signaling but also through indirect regulation of inflammatory tone and EV-mediated communication (Fabbrini et al., 2023).

The observed effects are highly relevant for neuropsychiatric and neurodegenerative conditions characterized by neurotransmitter imbalance, synaptic dysfunction, and chronic inflammation. By enhancing GABAergic and serotonergic pathways while suppressing inflammatory signaling, these probiotics may serve as adjunctive interventions in conditions such as anxiety, depression, autism spectrum disorder, and even neurodegenerative diseases like Alzheimer’s and Parkinson’s (Di Chiano et al., 2024). By enhancing GABAergic and serotonergic pathways while suppressing inflammatory signaling, these probiotics may serve as adjunctive interventions in conditions such as anxiety, depression, autism spectrum disorder, and neurodegenerative diseases like Alzheimer’s and Parkinson’s (Di Chiano et al., 2024). Although our study used *in vitro* models, the data suggest potential translational applications pending *in vivo* validation (e.g., gnotobiotic or humanized mice). Importantly, the strain-specific nature of the effects emphasizes the need for precision probiotic interventions tailored to individual neurochemical imbalances (Oroojzadeh et al., 2022).

### 4.1 Limitations and future directions

While this study provides robust *in silico* and *in vitro* validation, several limitations must be acknowledged. First, neuronal cell lines and iPSC-derived neurons may not fully recapitulate the complexity of *in vivo* neuroimmune interactions. Second, conditioned media experiments confirm the role of soluble metabolites but do not capture the impact of gut epithelial and immune system crosstalk. Third, although SPIEC-EASI networks revealed stable associations, causality between microbial metabolites and specific neuronal gene expression changes remains to be established.

Future work should employ *in vivo* models (e.g., gnotobiotic mice) and clinical trials to validate translational relevance. Integration of metabolomics, EV proteomics, and single-cell or spatial transcriptomics will further refine mechanistic understanding and cell-type-specific effects. Testing multi-strain consortia could reveal synergistic or antagonistic interactions beyond single-strain interventions.

## 5 Conclusion

In conclusion, this study highlights the power of multi-omics integration to uncover the mechanistic underpinnings of probiotic action on neuronal health. By incorporating n = 3 biological replicates, standardized MOI 1:10 exposures, validated baseline neurotransmitter measurements, and consistent gene nomenclature (IL6, TNF, GAD1), we demonstrate reproducible, strain-specific modulation of neurotransmitter pathways, synaptic signaling, and neuroinflammation. *L. rhamnosus* GG and *B. longum* 1714 exert complementary, strain-specific effects, modulating neurotransmitter pathways, synaptic signaling, and neuroinflammation. By linking microbial genomic features to host transcriptomic responses and validating them *in vitro*, our findings provide a compelling rationale for precision probiotic development targeting the gut–brain axis, with potential applications in mood regulation, cognitive resilience, and neuropsychiatric conditions.

## Data Availability

The original contributions presented in the study are included in the article/supplementary material. Further inquiries can be directed to the corresponding author.
